# Determination of *Alternaria* Toxins in Tomato, Wheat, and Sunflower Seeds by SPE and LC-MS/MS—A Method Validation Through a Collaborative Trial

**DOI:** 10.1093/jaoacint/qsab094

**Published:** 2021-07-22

**Authors:** Carlos Gonçalves, Ádam Tölgyesi, Katrien Bouten, Piotr Robouch, Hendrik Emons, Joerg Stroka

**Affiliations:** European Commission, Joint Research Centre (JRC), Geel, Belgium; Mertcontroll Ltd., Szabadság street 13, 2144 Kerepes, Hungary; European Commission, Joint Research Centre (JRC), Geel, Belgium; European Commission, Joint Research Centre (JRC), Geel, Belgium; European Commission, Joint Research Centre (JRC), Geel, Belgium; European Commission, Joint Research Centre (JRC), Geel, Belgium

## Abstract

**Background:**

*Alternaria* toxins are ubiquitous contaminants in highly consumed food products. Therefore, they are candidates to be regulated by EU legislation. In this context, the availability of reliable analytical methods is a keystone both for protecting the health of citizens and smooth functioning of the European market.

**Objective:**

This paper describes an advanced LC-MS/MS method based on isotope dilution quantification suitable for the determination of altenuene, alternariol, alternariol monomethyl ether, tenuazonic acid, and tentoxin in tomato puree, wheat, and sunflower seeds.

**Methods:**

The method has been validated in an interlaboratory study that included the analysis of both spiked and naturally contaminated food commodities. Twenty-three participants contributed with analytical data.

**Results:**

The average recoveries and relative standard deviations for repeatability and reproducibility obtained across the tested matrixes were: 97, 8.0, and 23%, for altenuene, respectively; 95, 9.2, and 17% for alternariol, respectively; 98, 6.4, and 13% for alternariol monomethyl ether, respectively; 97, 4.2, and 9.3% for tenuazonic acid, respectively; and 102, 5.6, and 15% for tentoxin, respectively. The method enabled the determination of all tested *Alternaria* toxins close to or below 1 µg/kg.

**Conclusion:**

Overall, the method showed a satisfactory trueness and precision, complying with the requirements for the monitoring of mycotoxins in food in the EU. It is currently under evaluation by the European Committee for Standardization for adoption as a standard method.

**Highlights:**

Isotope dilution mass spectrometry method for the determination of Alternaria toxins in food.

*Alternaria* species, the most prevalent being *Alternaria alternata*, produce more than 70 secondary metabolites, but only a few of them have been structurally elucidated and reported as relevant mycotoxins (1, 2). Grains and derived products, oil seeds and products thereof, tomato and tomato products, fruits and fruit products, beer, and wine are frequently contaminated with *Alternaria* toxins (ATs) (2, 3).

The ATs of major health concern are alternariol (AOH), alternariol monomethyl ether (AME), altenuene (ALT), tentoxin (TEN), and tenuazonic acid (TeA). AOH, AME, and ALT are dibenzo-α-pyrones, TEN is a cyclic tetrapeptide, and TeA is a tetramic acid derivative (3, 4). ALT and TeA have shown high acute toxicity in vitro and in animal experiments. AME and AOH are less acutely toxic, however, they have been described to induce mutagenic, carcinogenic, cytotoxic, and genotoxic effects (1, 3, 5–7).

The European Food Safety Authority (EFSA) reviewed the levels of ATs quantified in different food commodities in the period 2010–15 (8). The highest mean levels (upper bound) of AOH were observed in buckwheat (33.1 µg/kg) and oats (39.7 µg/kg). AOH was also quantified in tomato puree (17.1 µg/kg), tomato sauce (17.4 µg/kg), and sun-dried tomatoes (17.4 µg/kg). The highest mean levels of AME were found in tree nuts and oil seed samples, in particular chestnuts (17.5 µg/kg) and sesame seeds (11.8 µg/kg). TeA was predominantly present in tomatoes and tomato products. High levels were reported in dried tomato soup with a mean content of 351 µg/kg, sun-dried tomatoes (233 µg/kg), and tomato puree (212 µg/kg). TeA was also quantified in fresh tomatoes (54 µg/kg), in tomato sauce, tomato ketchup, and tomato juice. The highest levels of TEN were observed in sunflower seeds (82 µg/kg) where it was present in almost half of the samples.

Several other reviews on the occurrence of ATs in food have been published (2, 3, 9–11). The data gathered by Fraeyman et al. (9) revealed that grain samples were frequently contaminated with TeA (15–100% of the samples), followed by TEN (77%), AOH (2.4–31%), AME (3–26%), ALT (2.6–7%), and altertoxin‐I (2.4%). The TeA levels in unprocessed cereals reached more than 4200 µg/kg in wheat, while the maximum levels of AOH, AME, ALT, and TEN remained about one order of magnitude lower. TeA was found in more than 80% of the sunflower seed and oil samples with a maximum content of 1350 µg/kg. TeA was also detected in all tomato concentrates and almost all tomato sauces (78–100% of the samples), pastes (80%), and juices (50–100%) analyzed (maximum 100–462 µg/kg). Other ATs were also frequently detected in tomato products: AOH (28–86% of the samples), AME (20–78%), and TEN (21–64%). Tralamazza et al. (11) reviewed the publications of the period 2012–17 covering a broad range of staple foods (maize, wheat, barley, rice, sorghum, and soya). TeA scored the highest occurrence rate and average levels (58%, 140 µg/kg), followed by macrosporin (55%, 38 µg/kg), TEN (34%, 10 µg/kg), AME (37%, 34 µg/kg), and AOH (33%, 21 µg/kg) (11).

The occurrence of TeA in food led to an estimation of the 95th percentile dietary exposure of the population exceeding the threshold of toxicological concern by a factor of 1.4 (2.4 in the case of infants). Therefore, consumption of food contaminated with TeA raises also toxicological concerns, already declared for AOH and AME (6, 9).

The “gold standard” for the determination of ATs in food is by using LC-MS/MS. A number of review papers addressing approaches for the determination of ATs in food were published (5, 7, 12, 13). According to the data collated by Man et al. (7), only LC-MS/MS can provide LOQs around or below 1 µg/kg. Fulfilling this goal still depends on the target matrix and the type of extraction/concentration procedure performed upfront. A sample enrichment/cleanup step, mostly based on solid-phase extraction (SPE), is usually needed to analyze ATs in cereals, cereal products, oil seeds, and other solid samples at sub-µg/kg levels. Collections of best performing methods can be found in Man et al. (7) and Escrivá et al. (5).

Due to pronounced matrix effects, an accurate quantification of ATs in food can only be reasonably achieved by using matrix-matched calibrations or preferably stable isotope dilution procedures. Isotopically labelled ATs have been used in research studies during the past 10 years, but they were not available commercially before 2018 (14, 15). The method described here is the first for ATs that was tested in a interlaboratory study using isotopically labelled internal standards. Details on the method development and in-house validation can be found in Gonçalves et al. (16).

Despite the multitude of analytical methods and monitoring studies published in the last 5 years, no standardized analytical method is yet available. In the frame of the mandate M/520 from the European Commission to the European Committee for Standardization (CEN), within TC 275 WG 5 “Horizontal methods for Food—Biotoxins”, the Joint Research Centre (JRC) organized a ring-trial for the validation of an analytical method for the determination of AOH, AME, ALT, TeA, and TEN in cereals, tomato, and sunflower seeds at levels down to 1 µg/kg. This paper describes the proposed analytical protocol and the outcome of this validation study.

## Experimental

### Safety Precautions

The use of this protocol involves hazardous materials, operations, and equipment. This protocol does not address all the safety problems associated with its use. It is the responsibility of the user of this protocol to establish appropriate safety and health practices and to determine the applicability of regulatory limitations before use.

Some *Alternaria* toxins exhibit genotoxic and mutagenic effects.

Protective clothing, gloves, and safety glasses should be worn at all times, and all sample preparation steps should be carried out in a fume hood. The disposal of waste solvents should be carried out according to applicable environmental rules and regulations of the International Agency for Research on Cancer (IARC).

### Chemicals

Use only chemicals of recognized analytical grade and water complying with EN ISO 3696.

- *Nitrogen compressed ga*s.—Purity equivalent to *φ* = 99.99% or better.
- *Water.—*HPLC grade.
- *Water.—*LC-MS grade.
- *Methanol.—*Analytical grade.
- *Methanol.—*LC-MS grade.
- *Ethyl acetate.—*Analytical grade or higher.
- *Acetic acid.—* *ω* ≥ 99.7%.
- *Ammonium hydroxide.—*LC-MS grade, mass fraction *ω*(NH_4_OH) = 25%.
- *Ammonium acetate.—*LC-MS grade.
- *Polysorbate 20 (* *Tween^®^20)* *.—*Analytical grade.
- *Alternaria toxins’ standards.—*For example, crystalline, as a film or as a reference material.
  1. *Altenuene.—*At least *ω* = 96% purity.
  2. *Alternariol.—*At least *ω* = 96% purity.
  3. *Alternariol monomethyl ether.—*At least *ω* = 96% purity.
  4. *Tentoxin.—*At least *ω* = 96% purity.
  5. *Tenuazonic acid.—*At least *ω* = 96% purity.
- *Isotopically labelled internal standards.—*For example, crystalline or as a standard solution.
  1. *Altenuene isotopically labelled internal standard* *.—*E.g., ALT-(methoxy-d_3_, methyl-d_3_); ALT-d_6_.
  2. *Alternariol isotopically labelled internal standard* *.—*E.g., AOH-(methyl-d_3_); AOH-d_3_.
  3. *Alternariol monomethyl ether isotopically labelled internal standard* *.—*E.g., AME-(1-methyl-d_3_); AME-d_3_.
  4. *Tentoxin isotopically labelled internal standard* *.—*E.g., TEN-d_3_.
  5. *Tenuazonic acid isotopically labelled internal standard* *.—*E.g., TEA-(acetyl-^13^C_2_); TEA-^13^C_2_, mixture of diastereomers in methanol.

### Apparatus and Materials

Usual laboratory glassware and equipment and, in particular, the following:

- *pH meter.*
- *Polypropylene (PP) centrifuge tube*.—(50 mL) with scale on it.
- *Laboratory balanc*e.—Accuracy of 0.01 g.
- *Analytical balance.—*Accuracy of 0.01 mg.
- *Adjustable mechanical vertical or horizontal shaker.*
- *High speed blending device*.—E.g., Ultra-turrax^®^.
- *Centrifuge.—*With temperature control and capable of generating a relative centrifugal force of approximately 3200 *g*.
- *Graduated volumetric pipettes.—*10 mL capacity.
- *Displacement pipettes.—*10, 20, 100, 250, and 1000 µL capacity, with appropriate tips.
- *Solid-phase extraction (SPE) column.—*With hydrophilic modified styrene polymer with 6 mL reservoir capacity, 200 mg adsorbent mass, and 100 µm particle size or smaller. *Note:* Strata-XL from Phenomenex (Utrecht, the Netherlands) (6 mL, 200 mg, and 100 µm particle size) have shown to meet these specifications.
- *PP reservoirs* (approximately 25 mL).—Fit to SPE columns.
- *Polytetrafluoroethylene (PTFE) syringe filter*.—0.2 µm pore size and 13 or 15 mm diameter.
- *Syringe with needle.—*1 mL.
- *Vacuum manifold.—*For SPE clean up, with taps.
- *Mixer.—*With high shear rate (e.g., Vortex).
- *Sample concentrator.—*With temperature control and gas supply.
- *Glass receiving tubes.—*For sample elution and evaporation.
- *Silanized glass HPLC vials.—*Approximately 1.5 mL capacity and crimp caps or equivalent.
- *Volumetric flasks.—*5, 10, 50, 100, and 1 L capacity.
- *LC-MS/MS system.—*With the following components:
  1. *HPLC pumps.—*Capable of maintaining a binary gradient at flow rates appropriate for the analytical column in use with sufficient accuracy.
  2. *Degasser.—*Optional, for degassing HPLC mobile phases.
  3. *Injection system.—*Capable of injecting an appropriate volume of test solution with sufficient accuracy.
  4. *Column oven.—*Capable of maintaining a constant temperature.
  5. *HPLC reversed phase column*.—A suitable column providing sufficient retention capacity of the first eluting analyte (most polar). Three analytical columns proved to fulfil these requisites: XSelect^®^ HSS T3 (100 × 2.1 mm id, 2.5 µm particle size) and Acquity^®^ HSS T3 (100 × 2.1 mm id, 1.8 µm particle size) both from Waters (Milford, MA, USA); and Gemini^®^ NX-C18 (100 × 2.1 mm id, 3 µm particle size) from Phenomenex.
  6. *Pre-column.—*Recommended, with the same stationary phase material as the analytical column.
  7. *Triple stage mass spectrometer*.—Triple quadrupole, ion trap, or quadrupole linear ion trap instrument, amongst others, equipped with an electrospray ionization (ESI) interface and operated in multiple reaction monitoring (MRM) mode. Any ionization mode giving sufficient yield might be employed.
  8. *Computer-based instrument control and data evaluation system*.

### Solutions

- *Aqueous acetic acid solution* *φ = 1%.—*Add 10 mL acetic acid to a 1 L volumetric flask and dilute to volume with HPLC grade water.
- *Ammonium hydroxide solution* *ω = 2.3%.—*Add 1 mL ammonium hydroxide 25% to a 10 mL volumetric flask and dilute to volume with LC-MS grade water.
- *Ammonium acetate solution* ρ* = 1 mol/L.—*Dissolve 77.08 g ammonium acetate in 1 L of water.
- *Polysorbate 20 solution* *φ = 2%.—*Pipet 2 mL polysorbate 20 into a 100 mL volumetric flask and dilute to volume with water.
- *Extraction Solution.—*Prepare a mixture of methanol–water–acetic acid (85 + 14+1, by volume) in a 1 L volumetric flask.
- *Elution Solution.—*Prepare a mixture of methanol–ethyl acetate (75 + 25, by volume) in a 1 L volumetric flask.
- *HPLC mobile phase A.—*5 mmol/L ammonium acetate buffer solution at pH ∼8.0. Dilute 5 mL ammonium acetate solution to 1 L with LC-MS water. Adjust the pH to between 7.95 and 8.05 with ammonium hydroxide 2.3%.
- *HPLC mobile phase B.—*Methanol.
- *Standard solutions.*
  1. *Stock standard solutions of ALT, AOH, AME, TEN,* *and TeA.—*At a concentration of 100 µg/mL. Weigh accurately suitable amounts of crystalline powders of known purity or dissolve dried-down films according to the instructions provided by the supplier. We used the second option: dried-down films were reconstituted in 1.000 mL of methanol resulting in the concentration and uncertainty indicated on the certificate. The concentration of ALT in the commercial standard was 10 µg/mL.
  2. *Working standard solution 1.—*Containing ALT, AOH, and AME at a concentration of 500 ng/mL, TEN at a concentration of 2500 ng/mL, and TeA at a concentration of 5000 ng/mL. Dilute accurate volumes of stock standard solutions with methanol in a 5 mL volumetric flask.
  3. *Working standard solution 2* *.—*Containing ALT, AOH, AME at a concentration of 100 ng/mL, TEN at a concentration of 500 ng/mL, and TeA at a concentration of 1000 ng/mL. Dilute an accurate volume of working standard solution 1 with methanol in a 5 mL volumetric flask.
- *Internal standard solutions.*
  1. *Stock solutions of ALT-(methoxy-d_3_, methyl-d_3_), AOH-(methyl-d_3_), AME-(1-methyl-d_3_), TEN-d_3_* *,* *and TeA-(acetyl-^13^C_2_).—*For example, at a concentration of 750 µg/mL: dissolve 1.12 mg crystalline powder of each substance in 1500 µL methanol in individual vials. TeA-(acetyl-^13^C_2_) might be supplied already as a methanolic solution with a similar concentration. Other isotopologues with a degree of labelling sufficient to avoid interference from the native substance in mass spectrometry are also acceptable.
  2. *Internal standard solution 1.—*Containing AOH-(methyl-d_3_), AME-(1-methyl-d_3_), and TEN-d_3_ at a concentration of 5 µg/mL, ALT-(methoxy-d_3_, methyl-d_3_) at a concentration of 10 µg/mL and TeA-(acetyl-^13^C_2_) at a concentration of 25 µg/mL. Dilute accurate volumes of stock solutions with methanol in a 5 mL volumetric flask.
  3. *Internal standard solution 2.—*Containing AOH-(methyl-d_3_), AME-(1-methyl-d_3_), and TEN-d_3_ at a concentration of 500 ng/mL, ALT-(methoxy-d_3_, methyl-d_3_) at a concentration of 1000 ng/mL, and TeA-(acetyl-^13^C_2_) at a concentration of 2500 ng/mL in methanol. Dilute an accurate volume of solution internal standard solution 1 with methanol in a 10 mL volumetric flask.
*Note 1:* All solutions should be well homogenized after preparation. Commercially available solutions with equivalent properties to those listed may be used.
*Note 2:* If not stated otherwise, all reagents, samples, and equipment used in this protocol should be at room temperature before any kind of manipulation takes place.

### Standard Operating Procedure (SOP)

#### Principle

A test portion of the sample is spiked with the isotopically labelled internal standards and extracted with a methanol–water–acetic acid solution. The mixture is centrifuged and an aliquot of the supernatant is collected. The extract is diluted with an equal volume of 1% (v/v) aqueous acetic acid solution and concentrated on a polymeric SPE column. The extract is eluted from the SPE cartridge with a methanol and ethyl acetate mixture. The eluate is then evaporated, reconstituted, filtered, and analyzed by LC-MS/MS.

*Note:* The herein described protocol gives a general outline of the experimental steps and instrumental conditions required for the determination of ATs in tomato, wheat, and sunflower seeds. The participants in the interlaboratory study received a detailed SOP which they had to follow strictly. The implementation of the steps below require that the analyst is sufficiently experienced with SPE and LC-MS/MS.

#### Extraction

Weigh 2.00 g sample (duly homogenized) to the nearest 0.01 g into a 50 mL centrifuge tube. Add 100 µL internal standard solution 2. Add 14.0 mL extraction solution to tomato puree samples and 15.0 mL to wheat and sunflower seed samples. Extract the samples during 45 ± 1 min applying vigorous agitation in a wrist-type shaker. Centrifuge the sample for at least 10 min at approximately 3200 *g* and transfer 7.5 mL of the supernatant (equal to 1.0 g sample) into a new 50 mL centrifuge tube. Dilute with an equal volume of 1% (v/v) aqueous acetic acid solution.

#### Solid phase extraction/cleanup

Condition the SPE column by passing through 7 mL methanol, followed by 7 mL ultrapure water and 4 mL 1% (v/v) acetic acid solution. Close the tap under the column and pipet another 3 mL 1% (v/v) acetic acid solution into the SPE column. Load the diluted sample into the reservoir. Open the tap and let the extract pass through at a flow rate of approximately 1 drop per second. Remove the reservoir and wash the column with 4 mL 2% (v/v) polysorbate 20 solution followed by 4 mL 1% (v/v) aqueous acetic acid solution. Dry the column thoroughly under vacuum.

#### Sample test solution

Elute the extract with 7 mL elution solution into a glass tube. Evaporate the eluate to dryness at 50 °C under a gentle stream of nitrogen. Add 400 µL methanol to the glass tube and re-dissolve the residue using a vortex shaker. Add 600 µL HPLC mobile phase A and vortex again. Filter the extract through a PTFE syringe filter into an HPLC vial.

#### Calibration solutions

Prepare calibration solutions at five concentration levels: 1, 5, 10, 25, and 100 ng/mL of ALT, AOH, AME; 5, 25, 50, 125, and 500 ng/mL of TEN and 10, 50, 100, 250, and 1000 ng/mL of TeA. Results reported in ng/mL are taken as equivalent to µg/kg. To prepare 1 mL calibration solution, pipet suitable amounts of working standard solutions 1 and 2, 50 µL internal standard solution 2, 600 µL HPLC mobile phase A,and complete with methanol. Prepare a blank by mixing 600 µL mobile phase A and 400 µL methanol.

#### LC-MS/MS Analysis

#####  

**Figure 1.**
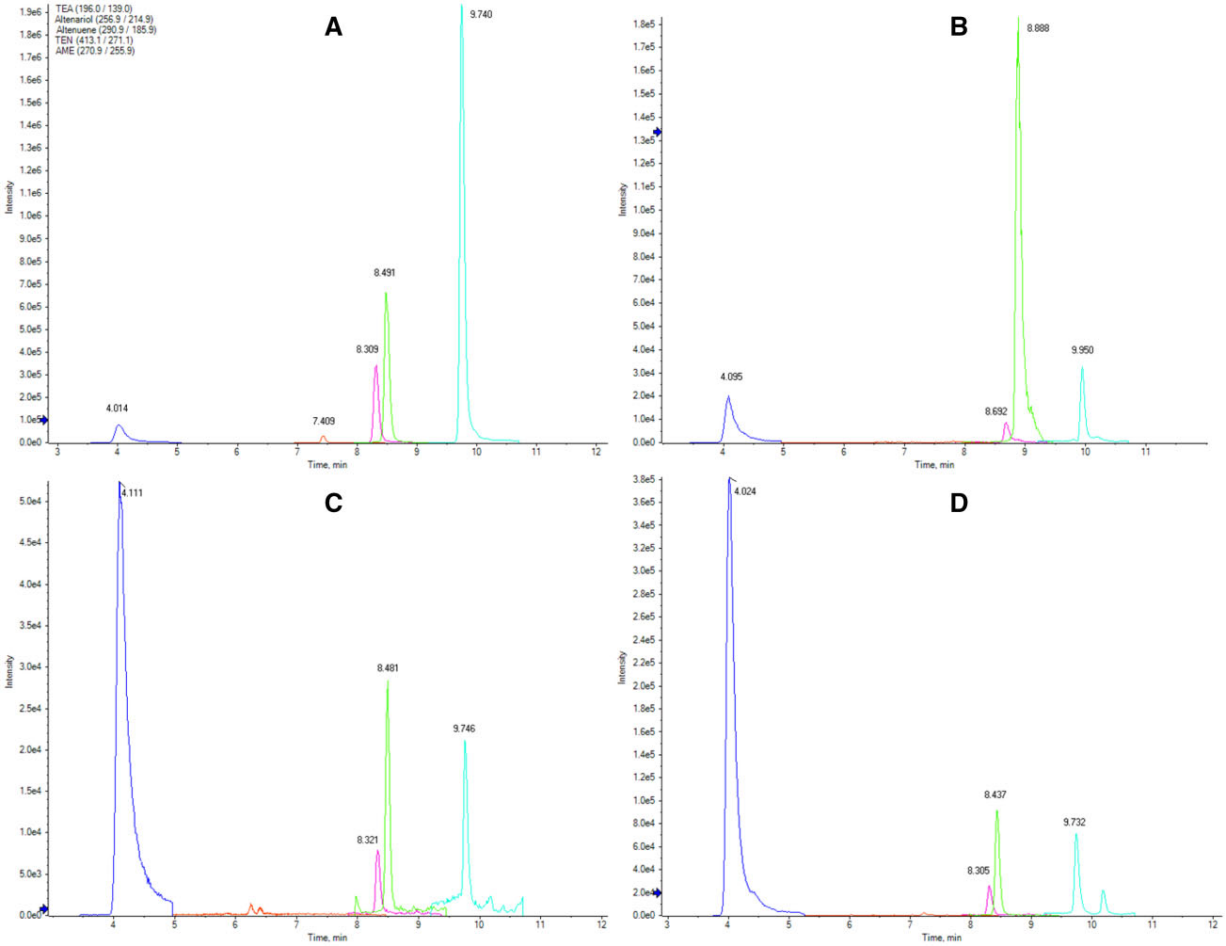
Extracted ion chromatograms of (A) a medium-level calibration standard, (B) tomato puree sample code Y, (C) wheat sample code G, and (D) sunflower sample code U. Retention times: TeA—4 min, ALT—7.4 min, AOH—8.3 min, TEN—8.5 min, and AME—9.7 min.

*Chromatographic conditions* The combination of analytical column, mobile phase composition, gradient settings, and injection volume should be such that it allows obtaining an acceptable chromatographic separation of the analytes and reliable results at the required levels, with sufficient selectivity. Acceptable chromatograms can be achieved using a column with a capacity factor of at least 3 (k′ ≥ 3.0) and a minimum plate number of 2000 (N ≥ 2000) for any of the analytes. The column Waters XSelect HSS T3 (100 × 2.1 mm id, 2.5 µm particle size) equipped with an XSelect HSS T3 VanGuard pre-column (5 × 2.1 mm id, 2.5 µm particle size) was employed during the in-house validation as it provided the best resolution (plate number for any analyte N ≥ 6890) and peak symmetry (0.92–1.39) at a lower system pressure (Figure 1A). The following conditions have been proven to offer the required instrumental performance: mobile phase A—5 mmol/L ammonium acetate buffer at about pH 8.0; mobile phase B—methanol; flow rate—0.3 mL/min; injection volume—5 µL; autosampler temperature of 10 °C; column oven temperature of 30 °C; gradient elution—start with 10% mobile phase B and maintain for 1 min, then raise to 100% mobile phase B until 10 min and maintain for 2 min, return to the initial conditions in 0.2 min and stabilize the gradient until 16 min. 

*Mass spectrometry conditions* ATs are weak acids that ionize efficiently in an alkaline environment generating deprotonated precursor ions ([M-H]^-^). The LC-MS/MS ion source temperatures (i.e., vaporizer, drying gas), gas flows, and voltages (i.e., capillary) depend on the instrument used for analysis and should be optimized in each laboratory. The instrument used in most of our experimental work was a Sciex QTrap 6500 for which the following conditions were found to be optimal: ion source—ESI; ionization mode—negative; detection—MS/MS in multiple reaction monitoring (MRM) mode; curtain gas—20 units; collision gas—high; ion spray voltage—4.0 kV; temperature of 600 °C; ion spray gas—1–30 units; and ion spray gas—2–30 units. Similarly, the optimal conditions for the MS/MS detection (i.e., collision energy, dwell times, and time segments) are also instrument-dependent and should be optimized on a case-by-case basis. Optimized MRM settings for the Sciex QTrap 6500 system are given in Table 1.

**Table 1.** Instrumental conditions set up on the Sciex QTrap 6500 mass spectrometer for the analysis of ATs. Acquisition time window in scheduled MRM—90 s.

Compounds	Time, min	Precursor ion [M − H]^−^, *m/z*	Product ion, *m/z*	DP^a^, V	EP^b^,V	CE^c^, V	CXP^d^, V
TeA	3.9	196.0	111.8	−55	−10	−32	−9
			139.0^e^	−55	−10	−26	−7
TeA-^13^C_2_		197.9	113.9	−20	−10	−34	−13
			140.8^e^	−20	−10	−28	−17
ALT	7.6	290.9	185.9^e^	−80	−10	−34	−11
			214.1	−80	−10	−28	−13
ALT-d_6_		297.0	189.0^e^	−90	−10	−38	−9
			216.9	−90	−10	−28	−13
AOH	8.4	256.9	212.0	−65	−10	−38	−9
			214.9^e^	−65	−10	−38	−19
AOH-d_3_		260.0	215.0	−65	−10	−40	−13
			217.9^e^	−65	−10	−38	−25
TEM	8.75	413.1	140.8	−65	−10	−24	−9
			271.1^e^	−65	−10	−20	−15
TEM-d_3_		416.1	141.0	−60	−10	−26	−9
			274.0^e^	−60	−10	−22	−17
AME	9.8	270.9	227.8	−60	−10	−38	−13
			255.9^e^	−60	−10	−28	−19
AME-d_3_		274.0	231.1	−60	−10	−38	−15
			258.8^e^	−60	−10	−30	−27

a DP = Declustering potential.

b EP = Entrance potential.

c CE = Collision energy.

d CXP = Collision cell exit potential.

e Suggested quantifier ion transitions.

**Table 2.** Performance data for measurements of ALT in tomato puree and wheat

Parameters	Tomato puree No. 1, nc^a^	Tomato puree No. 2, nc	Tomato puree No. 3, nc	Tomato puree No. 4, spk^b^	Tomato puree No. 5, spk	Wheat No. 1, nc	Wheat No. 2, nc	Wheat No. 3, spk	Wheat No. 4, spk	Wheat No. 5, spk
Pairing sample codes	P/Z	Lambda/N	M/R	Beta/Y	A/H	B/J	G/K	F/L	I/T	D/O
Number of laboratories	3	1	1	12	15	0	1	14	15	12
Number of non-compliant results	0	0	1	2	2	0	1	0	2	1
Number of accepted results	3	1	0	10	13	0	0	14	13	11
Robust mean value, $\bar{x}$, µg/kg	0.95	<1	<1	2.18	11.2	<1	<1	13.8	4.73	9.16
Repeatability standard deviation, *s*_r_, µg/kg				0.21	0.74			1.10	0.37	0.47
Relative repeatability standard deviation, RSD_r_, %				9.7	6.6			8.0	7.9	5.1
Repeatability limit, *r*, µg/kg				0.59	2.08			3.08	1.04	1.31
Reproducibility standard deviation, *s*_R_, µg/kg				0.41	1.86			2.97	1.48	2.94
Relative reproducibility standard deviation, *RSD*_R_, %				18.7	16.5			21.6	31.3	32.0
Reproducibility limit, *R,* µg/kg				1.14	5.19			8.33	4.14	8.22
HorRat value				0.9	0.8			1.0	1.4	1.5
Apparent recovery, %^c^				107.4	107.3			94.8	92.7	87.2
Target level, µg/kg				2.03	10.5			14.5	5.10	10.5

a nc = Naturally contaminated sample.

b spk = Spiked sample.

c Target mass fractions derived by formulation.

##### Injection sequence

Equilibrate the LC-MS/MS instrument by injecting blank solutions and matrix-free standard solutions until a stable response is obtained. Analyze the calibration solutions once at the beginning of the injection sequence. Analyze the blank solutions and the sample test solutions in a sequence that avoids carry over and cross-contamination. Analyze quality control standards and duplicates periodically, according to the laboratory’s internal quality system procedures.

##### Identification and quantification

Identify the presence of ATs in the sample test solution by following the provisions given below and laid down in the guidance document SANTE/12089/2016 (17). The retention time of the analyte in the chromatogram of the sample should correspond to the average retention time of that analyte in the calibration standards measured in the same sequence with a tolerance of ±0.2 min or ±50% of the peak width at half height (whichever is larger). The retention time of the analyte should correspond to that of its labelled internal standard with a tolerance of ±0.05 min. Calculate the ion ratios defined as the response of the ion peak with the lower area divided by the response of the ion peak with the higher area (relative intensity) from the calibration solutions. The ion ratios observed in the chromatograms of the test samples should match (±30% of tolerance) the respective average ion ratio in the calibration solutions from the same sequence.

The MRM ion transition with the largest signal-to-noise ratio (less interferences) should be selected as the quantifier ion trace. Choose the corresponding MRM transition of the internal standard, taking into account the degree of isotope labelling. The peak areas in the respective extracted ion chromatograms are used in all subsequent calculations.

Calculate the response ratio of each AT to the respective isotopically labelled internal standard. Plot the response ratio of the analyte in the calibration solutions against the respective mass fractions (µg/kg) and compute the calibration parameters using a linear regression model. The mass fractions of the ATs in the sample are directly obtained interpolating the response ratio of the analytes observed in the chromatogram of the sample test solution in the above calibration.

For the quantification of levels of analytes with a response ratio between the first two calibration levels, it is essential to ensure a good fit of the calibration line by forcing it through the origin or by employing a weighing factor of 1/*x* or 1/*x*^2^, whichever leads to a smaller offset.

If the amount of ATs exceeds the working range, the sample should be reanalyzed by weighing a second portion of 2.00 g and adding a multiple of the volumes of extraction solution and internal standard solution 2. Take that into consideration in the subsequent calculations.

### Preparation of the Test Materials

The target matrixes for this study were tomato puree, wheat, and sunflower seeds. Three naturally contaminated materials (or blends thereof) and two spiked test materials of each matrix were prepared for the interlaboratory study. The levels of each AT were planned to spread over the working range of interest. The spiking levels were chosen to achieve mass fractions of ATs relevant for validation but not available in the naturally incurred materials.

Sunflower seeds and tomato puree containing low amounts of ATs were acquired in the local retail market. Highly contaminated sunflower seed meal (kernel and husks) and tomatoes with visible black fungi infestations were used to modulate the levels of ATs in the materials. Sunflower seeds were cryo-milled (Palla VM-KT, Cologne, Germany) leading to flours with a particle size <0.5 µm, blended in pre-defined amounts to obtain the desired levels and then homogenized under cooling conditions. Similarly, suitable amounts of various tomato products including the spoiled ones were blended and homogenized for 2 h with a propeller mixer (IKA Werke GmbH, Staufen, Germany). Two naturally contaminated wheat flours from the JRC stock each constituted a test material and a third (all with particle size <0.5 µm) was additionally spiked with ALT, AOH, and AME.

Blank tomato puree was prepared from fresh tomatoes. Approximately 6.5 kg tomatoes of good quality were washed, dipped in boiling water for 2 min, and peeled. Next, the pulp was minced in a Thermomix TM-31–1 apparatus (Vorwerk Elektrowerke, Wuppertal, Germany) and the seeds were removed by sieving the puree through a 1.25 mm pore size stainless-steel sieve. About 3.7 kg puree was obtained and further homogenized using a propeller. It was not possible to source blank sunflower seed samples, the lowest contaminated material contained about 146 µg/kg of TeA. The blank tomato puree, blank wheat, and low-level sunflower seeds were spiked at two levels each (low and high) to provide measurable levels of all five ATs. Amounts of 1.5 kg of the spiked materials were prepared. The blank tomato puree was directly spiked with appropriate volumes of standards and thoroughly mixed. The spiked sunflower seed materials were prepared by adding the required amounts of standards to 10% of sunflower powder previously defatted with hexane. This portion was then homogenized with the remaining material under cryogenic conditions. Defatting ensured that a very fine free-flowing powder was obtained carrying the spiked analytes that was then evenly dispersed. Methanol used as solvent for spiking was allowed to evaporate in the fume hood over three days. The spiked wheat materials were obtained by preparing a slurry of the flour in methanol–water (80+20, v/v) to which the ATs have been added. The ratio of the spiking solution to wheat flour was about 1:1 (volume/mass). After thorough mixing in an industrial bowl with paddle, the paste was spread on two trays and freeze-dried under a controlled program over 2 days. The blocks were then crunched and cryo-milled. The design and execution of the spiking were performed at our best technical capabilities to ensure the integrity, quantitative transfer, and homogeneous distribution of the ATs on the materials. Their water content was determined before and after the spiking process to correct the obtained mass fractions of the ATs for possible moisture variations.

The tomato puree materials were distributed in 50 mL falcons in portions of approximately 10 g. Similarly, the wheat and sunflower materials were put into 50 mL amber glass bottles and tightly closed with a screw cap featuring a Teflon lined septa and break-ring. The materials were immediately stored at −18 °C until dispatch. Each test material was coded in blind duplicates (e.g., E54 and V38) composed of about 75 units each. In total, 30 test samples (i.e., three matrixes, five contamination levels, blind duplicates) were produced for the interlaboratory study. The levels of ATs spiked onto the materials were considered as the reference mass fractions for the calculation of the recoveries. The mass fractions in the naturally contaminated materials were determined experimentally during the homogeneity assessment applying the SOP described herein and a fully gravimetric preparation of the calibration standards. The levels present in each material are provided in the *Results and Discussion* section.

### Homogeneity and Stability of the Samples

For assessing the homogeneity of the test materials, ten units of each of the 15 materials were randomly selected from the production batch. Two independent determinations were performed per container using the LC-MS/MS method described in the *SOP* section. The sequence of measurements was randomized. Homogeneity was evaluated according to ISO Guide 35:2017 (18). All materials proved to be adequately homogeneous regarding their content in ATs. Material wheat level 2 (codes G/K) had to be milled to pass a sieve of 0.12 mm pore size to ensure homogeneity of AOH. The raw data of the homogeneity and stability studies is provided as Supplemental Table S1.

The assessment of the stability of the test materials was carried out according to an isochronous experimental design (18, 19). The stability was investigated at 4 °C (7 and 14 days, short term) and −18 °C (7, 14, and 59 days, long term), whereas −70 °C was chosen as the reference temperature for sample storage. Two materials per matrix (one naturally contaminated and one spiked) underwent a stability check. The stability data obtained was evaluated according to the requirements of ISO Guide 35:2017 (18). A linear regression was calculated for each analyte and tested temperature over the duration of the study. The slopes of the linear regressions did not deviate significantly from zero in any of the cases, at 95% confidence level, hence the materials were declared stable at 4 °C and −18 °C. Previous works corroborate the stability of the ATs in solvent and tomato juice during several months (20, 21). To ensure that the temperature of the test materials would not exceed 4 °C during transport, the samples were dispatched in polystyrene boxes filled with dry ice.

### Interlaboratory Study

The participants in the validation study received the following materials:

- Thirty coded test materials for direct analysis (ten materials per matrix);
- working standard solutions 1 and 2 for calibration;
- internal standard solution 2;
- 35 Strata-XL SPE cartridges;
- 25 syringe filters.

Documentation and technical support were provided to enable them to implement the method strictly. It included a detailed SOP, a list of critical steps, and an accompanying letter where instructions for sample storage, analysis, and reporting were provided. The participants were given 7 weeks to report the results. They were instructed to analyze all the samples of one matrix on the same day and just one aliquot per bottle. They were also asked to provide chromatograms of the samples coded Y (tomato), D (wheat), and Q (sunflower).

All the results reported by the laboratories that have deviated from the SOP under investigation were systematically rejected. Left-censored values (<LOQ) could not be included in the statistical treatment. The remaining dataset was checked for consistency according to Mandel's *h and k* statistics (22). The resulting subset was used to derive the robust mean and the method performance parameters, namely the recoveries and the relative standard deviations for repeatability and reproducibility (RSD_r_ and RSD_R_, respectively). ProLab software (QuoData GmbH, Dresden, Germany) was used to perform the statistical treatment according to the algorithm A + S described in ISO 5725–5 (23). The recoveries were calculated by the ring-trial organizer. A fit-for-purpose relative standard deviation for reproducibility of 22% was derived from the modified Horwitz equation (24) to compute the Horwitz ratios (HorRat) (25).

## Results and Discussion

### Interlaboratory Method Validation

From the 30 laboratories that registered to the interlaboratory study, 23 returned analytical results in due time. The participants represented a cross-section of research, private, and official control laboratories from 11 EU Member States, the United Kingdom, and Switzerland.

Seven laboratories employed a Waters Acquity HSS T3, 1.8 µm column for analyzing the distributed samples; two used the equivalent Waters XSelect HSS T3, 2.5 µm column; five used a Phenomenex Gemini NX-C18, 5 µm column; three used a Waters Acquity UPLC BEH, 1.7 µm column; two used a Supelco Ascentis Express C18, 2.7 µm column, and three laboratories used other C18 and biphenyl columns. Figure 1 displays chromatograms generated in-house using a Waters XSelect HSS column for a calibration standard and samples of each of the three studied matrixes. The data submitted by the participants are compiled in Supplemental Table S2. Graphical plots of the normalized data pairs are shown in Supplemental Figures S1A–S1E.

The results reported by three laboratories were rejected due to the following technical reasons:

1. The extracted ion chromatograms of the samples coded D, Q, and Y provided by laboratory LC0022 displayed broad and split peaks for all analytes. This effect was also visible in the chromatogram of the highest calibration standard. Furthermore, this laboratory used an Orbitrap mass spectrometer in full-scan mode, differing from the mandatory mass spectrometry settings.
2. Laboratory LC0021 used an atmospheric pressure chemical ionization (APCI) source instead of the required ionization mode (ESI), while still using a triple quadrupole mass spectrometer. However, the results reported fell within the data range of the remaining participants, indicating that APCI could potentially be applied as ionization mode provided that the laboratories can a priori demonstrate an acceptable in-house performance.
3. Laboratory LC0010 faced some sensitivity issues and informed the study coordinator that their “results can only be regarded as indicative and semi-quantitative”.

The consistency check performed on the remaining data sets according to Mandel’s *h* and *k* statistics did not provide evidence to dismiss the whole dataset of a specific laboratory for any analyte (*see*  Supplemental Table S3).

Whenever less than eight valid datasets were submitted (mostly for very low contamination levels), no statistical parameters were calculated (26). For most of the analyte/matrix combinations 18–20 datasets were available for statistical treatment. A compilation of the mean mass fractions, precision, and trueness parameters is presented in Tables 2–11.

**Table 3.** Performance data for measurements of ALT in sunflower seeds

Parameters	Sunflower No. 1, nc^a^	Sunflower No. 2, nc	Sunflower No. 3, nc	Sunflower No. 4, spk^b^	Sunflower No. 5, spk
Pairing sample codes	E/V	S/W	Alfa/U	C/X	Miu/Q
Number of laboratories	2	3	2	8	14
Number of non-compliant results	1	1	1	2	2
Number of accepted results	1	2	1	6	12
Robust mean value, $\bar{x}$, µg/kg	<1	<1	<1	2.49	10.2
Repeatability standard deviation, *s*_r_, µg/kg					1.09
Relative repeatability standard deviation, *RSD*_r_, %					10.7
Repeatability limit, *r*, µg/kg					3.06
Reproducibility standard deviation, *s*_R_, µg/kg					2.08
Relative reproducibility standard deviation, *RSD*_R_, %					20.3
Reproducibility limit, *R,* µg/kg					5.81
HorRat value					0.9
Apparent recovery, %^c^					94.1
Target level, µg/kg				2.25	10.8

a nc = Naturally contaminated sample.

b spk = Spiked sample.

c Target mass fractions derived by formulation.

**Table 4.** Performance data for measurements of AOH in tomato puree and wheat

Parameters	Tomato puree No. 1, nc^a^	Tomato puree No. 2, nc	Tomato puree No. 3, nc	Tomato puree No. 4, spk^b^	Tomato puree No. 5, spk	Wheat No. 1, nc	Wheat No. 2, nc	Wheat No. 3, spk	Wheat No. 4, spk	Wheat No. 5, spk
Pairing sample codes	P/Z	Lambda/N	M/R	Beta/Y	A/H	B/J	G/K	F/L	I/T	D/O
Number of laboratories	22	22	21	16	22	6	13	22	18	21
Number of non-compliant results	4	4	3	3	4	1	0	3	2	3
Number of accepted results	18	18	18	13	18	5	13	19	16	18
Robust mean value, $\bar{x}$, µg/kg	27.4	12.9	6.06	1.82	9.68	2.04	1.83	46.7	4.90	14.4
Repeatability standard deviation, *s*_r_, µg/kg	1.70	0.66	0.49	0.09	0.61		0.27	3.35	0.40	1.38
Relative repeatability standard deviation, *RSD*_r_, %	6.2	5.1	8.1	4.7	6.3		14.5	7.2	8.2	9.6
Repeatability limit, *r*, µg/kg	4.77	1.84	1.37	0.24	1.70		0.74	9.37	1.13	3.87
Reproducibility standard deviation, *s*_R_, µg/kg	3.45	2.01	0.86	0.26	1.33		0.50	7.09	0.66	2.75
Relative reproducibility standard deviation, *RSD*_R_, %	12.6	15.6	14.2	14.2	13.8		27.1	15.2	13.5	19.2
Reproducibility limit, *R,* µg/kg	9.67	5.64	2.41	0.72	3.73		1.39	19.8	1.85	7.69
HorRat value	0.6	0.7	0.6	0.6	0.6		1.2	0.7	0.6	0.9
Apparent recovery, %^c^	100.1	98.0	104.5	91.0	96.7		105.2	94.9	95.9	92.9
Target level, µg/kg	27.3	13.2	5.80	2.00	10.0	2.10	1.74	49.2	5.11	15.5

a nc = Naturally contaminated sample.

b spk = Spiked sample.

c Target mass fractions derived by formulation, in the spiked samples, and assigned by the organizer, in the naturally contaminated samples.

**Table 5.** Performance data for measurements of AOH in sunflower seeds

Parameters	Sunflower No. 1, nc^a^	Sunflower No. 2, nc	Sunflower No. 3, nc	Sunflower No. 4, spk^b^	Sunflower No. 5, spk
Pairing sample codes	E/V	S/W	Alfa/U	C/X	Miu/Q
Number of laboratories	21	22	21	16	21
Number of non-compliant results	2	3	3	0	3
Number of accepted results	19	19	18	16	18
Robust mean value, $\bar{x}$, µg/kg	31.9	15.2	5.37	1.97	8.47
Repeatability standard deviation, *s*_r_, µg/kg	3.50	1.53	0.73	0.24	0.99
Relative repeatability standard deviation, *RSD*_r_, %	11.0	10.1	13.6	12.4	11.6
Repeatability limit, *r*, µg/kg	9.80	4.30	2.04	0.68	2.76
Reproducibility standard deviation, *s*_R_, µg/kg	4.66	2.90	1.25	0.35	1.69
Relative reproducibility standard deviation, *RSD*_R_, %	14.6	19.1	23.3	18.0	20.0
Reproducibility limit, *R,* µg/kg	13.1	8.13	3.50	0.99	4.74
HorRat value	0.7	0.9	1.1	0.8	0.9
Apparent recovery, %^c^	92.1	86.8	95.6	95.6	82.2
Target level, µg/kg	34.6	17.5	5.62	2.06	10.3

a nc = Naturally contaminated sample.

b spk = Spiked sample.

c Target mass fractions derived by formulation, in the spiked samples, and assigned by the organizer, in the naturally contaminated samples.

**Table 6.** Performance data for measurements of AME in tomato puree and wheat

Parameters	Tomato puree No. 1, nc^a^	Tomato puree No. 2, nc	Tomato puree No. 3, nc	Tomato puree No. 4, spk^b^	Tomato puree No. 5, spk	Wheat No. 1, nc	Wheat No. 2, nc	Wheat No. 3, spk	Wheat No. 4, spk	Wheat No. 5, spk
Pairing sample codes	P/Z	Lambda/N	M/R	Beta/Y	A/H	B/J	G/K	F/L	I/T	D/O
Number of laboratories	22	22	23	22	23	5	18	23	22	22
Number of non-compliant results	3	3	3	2	3	1	2	4	3	3
Number of accepted results	19	19	20	20	20	4	16	19	19	19
Robust mean value, $\bar{x}$, µg/kg	16.4	6.19	2.69	1.98	9.74	0.55	1.29	47.2	4.87	14.8
Repeatability standard deviation, *s*_r_, µg/kg	0.66	0.32	0.10	0.09	0.29		0.21	1.52	0.16	0.57
Relative repeatability standard deviation, *RSD*_r_, %	4.0	5.1	3.6	4.5	3.0		16.5	3.2	3.3	3.9
Repeatability limit, *r*, µg/kg	1.85	0.88	0.27	0.25	0.82		0.60	4.27	0.45	1.60
Reproducibility standard deviation, *s*_R_, µg/kg	1.89	0.68	0.32	0.26	0.86		0.34	5.04	0.51	1.12
Relative reproducibility standard deviation, *RSD*_R_, %	11.6	10.9	11.8	12.9	8.9		26.6	10.7	10.6	7.6
Reproducibility limit, *R,* µg/kg	5.30	1.90	0.89	0.72	2.42		0.96	14.1	1.44	3.13
HorRat value	0.5	0.5	0.5	0.6	0.4		1.2	0.5	0.5	0.3
Apparent recovery, %^c^	97.3	108.0	101.9	101.0	97.6		108.4	94.3	96.2	93.0
Target level, µg/kg	16.9	5.73	2.64	1.96	10.0	1.17	1.19	50.1	5.06	15.9

a nc = Naturally contaminated sample.

b spk = Spiked sample.

c Target mass fractions derived by formulation, in the spiked samples, and assigned by the organizer, in the naturally contaminated samples.

**Table 7.** Performance data for measurements of AME in sunflower seeds

Parameters	Sunflower No. 1, nc^a^	Sunflower No. 2, nc	Sunflower No. 3, nc	Sunflower No. 4, spk^b^	Sunflower No. 5, spk
Pairing sample codes	E/V	S/W	Alfa/U	C/X	Miu/Q
Number of laboratories	22	22	22	19	22
Number of non-compliant results	2	2	3	1	2
Number of accepted results	20	20	19	18	20
Robust mean value, $\bar{x}$, µg/kg	12.2	7.83	3.74	2.08	17.1
Repeatability standard deviation, *s*_r_, µg/kg	1.16	0.45	0.43	0.21	1.08
Relative repeatability standard deviation, *RSD*_r_, %	9.5	5.8	11.5	9.9	6.3
Repeatability limit, *r*, µg/kg	3.24	1.27	1.21	0.58	3.02
Reproducibility standard deviation, *s*_R_, µg/kg	1.75	1.08	0.57	0.39	1.75
Relative reproducibility standard deviation, *RSD*_R_, %	14.3	13.8	15.2	18.7	10.3
Reproducibility limit, *R,* µg/kg	4.90	3.01	1.59	1.09	4.91
HorRat value	0.7	0.6	0.7	0.8	0.7
Apparent recovery, %^c^	96.7	101.8	91.0	99.0	84.3
Target level, µg/kg	12.6	7.69	4.11	2.10	20.2

a nc = Naturally contaminated sample.

b spk = Spiked sample.

c Target mass fractions derived by formulation, in the spiked samples, and assigned by the organizer, in the naturally contaminated samples.

**Table 8.** Performance data for measurements of TeA in tomato puree and wheat

Parameters	Tomato puree No. 1, nc^a^	Tomato puree No. 2, nc	Tomato puree No. 3, nc	Tomato puree No. 4, spk^b^	Tomato puree No. 5, spk	Wheat No. 1, nc	Wheat No. 2, nc	Wheat No. 3, nc	Wheat No. 4, spk	Wheat No. 5, nc, spk^c^
Pairing sample codes	P/Z	Lambda/N	M/R	Beta/Y	A/H	B/J	G/K	F/L	I/T	D/O
Number of laboratories	22	22	22	23	23	23	22	21	22	23
Number of non-compliant results	4	4	4	4	4	4	3	2	3	4
Number of accepted results	18	18	18	19	19	19	19	19	19	19
Robust mean value, $\bar{x}$, µg/kg	961	499	183	47.0	194	265	162	41.8	55.1	413
Repeatability standard deviation, *s*_r_, µg/kg	32.3	18.0	4.59	1.93	6.99	14.1	9.47	2.26	1.72	9.73
Relative repeatability standard deviation, *RSD*_r_, %	3.4	3.6	2.5	4.1	3.6	5.3	5.9	5.4	3.1	2.4
Repeatability limit, *r*, µg/kg	90.5	50.4	12.9	5.40	19.6	39.5	26.5	6.33	4.80	27.3
Reproducibility standard deviation, *s*_R_, µg/kg	58.6	39.8	11.5	4.88	16.1	19.7	14.4	3.63	4.72	30.4
Relative reproducibility standard deviation, *RSD*_R_, %	6.1	8.0	6.3	10.4	8.3	7.4	8.9	8.7	8.6	7.4
Reproducibility limit, *R,* µg/kg	164	111	32.2	13.7	45.1	55.0	40.3	10.2	13.2	85.2
HorRat value	0.3	0.4	0.3	0.5	0.4	0.3	0.4	0.4	0.4	0.3
Apparent recovery, %^d^	96.7	94.0	95.4	94.5	97.5	89.1	101.1	113.4	106.0	100.5
Target level, µg/kg	994	530	192	49.8	199	297	160	36.8	52.0	411

a nc = Naturally contaminated sample.

b spk = Spiked sample.

c nc, spk = Naturally contaminated sample, additionally spiked.

d Target mass fractions derived by formulation, in the spiked samples, and assigned by the organizer, in the naturally contaminated samples.

**Table 9.** Performance data for measurements of TeA in sunflower

Parameters	Sunflower No. 1, nc^a^	Sunflower No. 2, nc	Sunflower No. 3, nc	Sunflower No. 4, nc	Sunflower No. 5, nc, spk^b^
Pairing sample codes	E/V	S/W	Alfa/U	C/X	Miu/Q
Number of laboratories	23	23	23	22	23
Number of non-compliant results	4	4	4	3	4
Number of accepted results	19	19	19	19	19
Robust mean value, $\bar{x}$, µg/kg	1618	936	488	147	198
Repeatability standard deviation, *s*_r_, µg/kg	70.1	44.0	21.4	5.96	13.7
Relative repeatability standard deviation, R*SD*_r_, %	4.3	4.7	4.4	4.1	6.9
Repeatability limit, *r*, µg/kg	196	123	59.8	16.7	38.2
Reproducibility standard deviation, *s*_R_, µg/kg	182	127	70.5	12.8	23.1
Relative reproducibility standard deviation, *RSD*_R_, %	11.2	13.6	14.5	8.7	11.7
Reproducibility limit, *R,* µg/kg	509	356	197	35.8	64.7
HorRat value	0.5	0.6	0.7	0.4	0.5
Apparent recovery, %^c^	89.3	92.5	88.6	100.4	97.0
Target level, µg/kg	1813	1011	550	146	204

a nc = Naturally contaminated sample.

b nc, spk = Naturally contaminated sample, additionally spiked.

c Target mass fractions assigned by the organizer.

**Table 10.** Performance data for measurements of TEN in tomato puree and wheat

Parameters	Tomato puree No. 1, nc^a^	Tomato puree No. 2, nc	Tomato puree No. 3, nc	Tomato puree No. 4, spk^b^	Tomato puree No. 5, spk	Wheat No. 1, nc	Wheat No. 2, nc	Wheat No. 3, nc	Wheat No. 4, spk	Wheat No. 5, nc, spk^c^
Pairing sample codes	P/Z	Lambda/N	M/R	Beta/Y	A/H	B/J	G/K	F/L	I/T	D/O
Number of laboratories	6	6	4	24	24	22	21	20	22	22
Number of non-compliant results	2	2	1	4	4	4	4	3	4	4
Number of accepted results	4	4	3	20	20	18	17	17	18	18
Robust mean value, $\bar{x}$, µg/kg	0.88	0.57	0.49	44.9	218	52.2	5.29	9.36	31.2	86.8
Repeatability standard deviation, *s*_r_, µg/kg				1.02	8.31	3.08	0.30	0.41	1.41	6.37
Relative repeatability standard deviation, *RSD*_r_, %				2.3	3.8	5.9	5.7	4.4	4.5	7.3
Repeatability limit, *r*, µg/kg				2.85	23.3	8.64	0.85	1.16	3.96	17.8
Reproducibility standard deviation, *s*_R_, µg/kg				4.21	34.4	6.68	1.31	2.29	3.80	14.7
Relative reproducibility standard deviation, *RSD*_R_, %				9.4	15.8	12.8	24.8	24.4	12.2	17.0
Reproducibility limit, *R,* µg/kg				11.8	96.2	18.7	3.67	6.40	10.6	41.3
HorRat value				0.4	0.7	0.6	1.1	1.1	0.6	0.8
Apparent recovery, %^d^				103.8	103.6	94.6	93.1	115.6	95.8	111.7
Target level, µg/kg				43.3	210	55.2	5.68	8.10	32.6	77.7

a nc = Naturally contaminated sample.

b spk = Spiked sample.

c nc, spk = Naturally contaminated sample, additionally spiked.

d Target mass fractions derived by formulation, in the spiked samples, and assigned by the organizer, in the naturally contaminated samples.

**Table 11.** Performance data for measurements of TEN in sunflower seeds

Parameters	Sunflower No. 1, nc^a^	Sunflower No. 2, nc	Sunflower No. 3, nc	Sunflower No. 4, spk^b^	Sunflower No. 5, spk
Pairing sample codes	E/V	S/W	Alfa/U	C/X	Miu/Q
Number of laboratories	24	24	23	24	24
Number of non-compliant results	4	4	4	4	4
Number of accepted results	20	20	19	20	20
Robust mean value, $\bar{x}$, µg/kg	75.2	38.9	19.2	47.8	168
Repeatability standard deviation, *s*_r_, µg/kg	3.51	2.09	0.56	5.59	14.7
Relative repeatability standard deviation, *RSD*_r_, %	4.7	5.4	2.9	11.7	8.7
Repeatability limit, *r*, µg/kg	9.83	5.84	1.57	15.7	41.1
Reproducibility standard deviation, *s*_R_, µg/kg	7.80	3.93	1.89	6.30	26.8
Relative reproducibility standard deviation, *RSD*_R_, %	10.4	10.1	9.8	13.2	15.9
Reproducibility limit, *R,* µg/kg	21.9	11.0	5.29	17.7	74.9
HorRat value	0.5	0.5	0.4	0.6	0.7
Apparent recovery, %^c^	107.7	108.2	101.6	97.7	84.2
Target level, µg/kg	69.8	36.0	18.9	49.0	200

a nc = Naturally contaminated sample.

b spk = Spiked sample.

c Target mass fractions derived by formulation, in the spiked samples, and assigned by the organizer, in the naturally contaminated samples.

**Figure 2.**
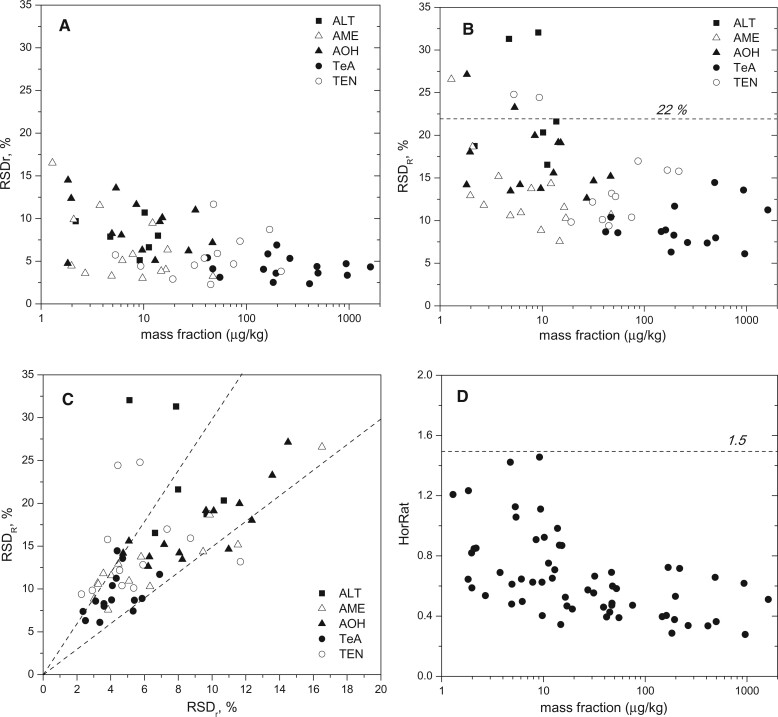
Plot of the method’s relative standard deviations for (A) repeatability, (B) reproducibility, and (D) HorRats as function of the mass fraction of the analytes in the samples. (C) Plot of the RSD_R_ as a function of the RSD_r_ for the five ATs determined in the 15 test materials. The lower and upper dashed lines delimit the region between the ratios 1.5 and 3.

Better precisions were obtained for measurements of TEN and TeA than for ALT and AOH, which can be partially explained by their higher mass fractions in the samples, as shown in Figure 2A and B. At first sight, AME seems to be an exception to this pattern, as the mass fractions are similar to those of ALT and AOH, but the precisions are closer to those of TeA and TEN. This can be explained by the lower LOQ achieved for AME. The following LOQs were determined in the frame of the in-house validation: 0.2, 0.35, 0.9, 1.4, and 2.6 µg/kg for AME, AOH, TEN, ALT, and TEA, respectively.

**Figure 3.**
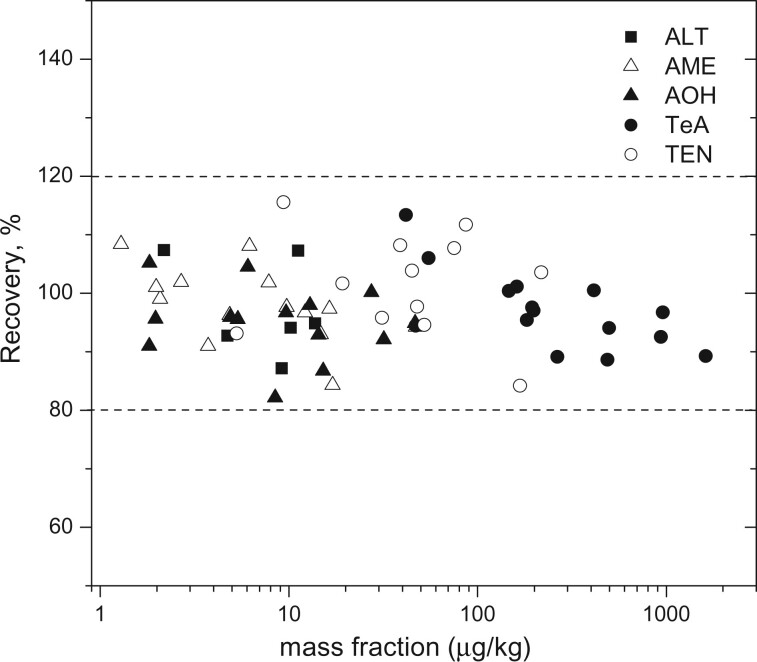
Plot of the method’s recoveries as function of the mass fraction of the analytes in the samples.

Furthermore, the following conclusions can be drawn:

1. The relative standard deviations for repeatability (RSD_r_) are mostly below 15% (Figure 2A) with only one single exception, while those for reproducibility (RSD_R_) are mostly below 22%, except for some mass fractions below 10 µg/kg (Figure 2B).
2. The ratios RSD_R_/RSD_r_ range between 1.5 and 3 for most of the measurands investigated, as indicated in Figure 2C. This is similar to that reported in the CEN/TS 17174 document (27).
3. All HorRat values are below 1.5 for all the investigated measurands (Figure 2D). Hence, the precision parameters obtained in this interlaboratory study are better than those obtained in a previous study conducted when no isotopically labelled internal standards were commercially available for any AT (28). Therein, some HorRat values for AME and TeA were above 2 since the matrix effects could not be appropriately compensated using matrix-matched calibrations under routine conditions.
4. Satisfactory recovery values ranging from 82 to 116% were obtained for all ATs in all matrixes (Figure 3). The recoveries determined herein express the apparent recovery of the analytes, since the SOP prescribes the addition of the internal standard at the beginning of the sample preparation. In addition, no significant difference was observed between the recovery values calculated for spiked samples and in naturally incurred samples. Furthermore, no trends could be identified based on the contamination level analyzed or the matrix investigated. These observations enable the use of calibrations with substances in neat solvent to quantify the ATs in food samples. This is highly advantageous as: (*i*) it reduces the laboratory work; (*ii*) it avoids the need to source strictly blank matrixes, which are difficult to find for some analytes; (*iii*) it pollutes the LC-MS/MS instrument less compared with the analysis of matrix-matched calibration standards; and (*iv*) it requires only one single calibration for the different matrixes.

## Conclusions

The first international interlaboratory study for the validation of an LC-MS/MS method for the determination of ATs in tomato, wheat, and sunflower seeds was successfully conducted.

The technical improvements introduced in the LC-MS/MS method, namely the use of isotopically labelled internal standards, proved to be crucial for delivering performance parameters fully in compliance with the requirements for the analysis of mycotoxins.

HorRat ratios below 1.5 and recoveries between 82 and 116% were obtained for all measurements of ATs in all the tested matrixes. The performance characteristics obtained demonstrate that the proposed method is suitable for the determination of mycotoxins in food (29). Hence, this method seems to be a good candidate for standardization.

## Supplemental Information

Supplemental information is available on the *J. AOAC Int*. website.

## CRediT Author Statement

###  

Goncalves, Carlos: Data curation (Lead), Formal analysis (Equal), Investigation (Equal), Methodology (Equal), Project administration (Lead), Writing—original draft (Lead). Tolgyesi, Adam: Formal analysis (Equal), Investigation (Equal), Methodology (Equal), Validation (Equal), Writing—review & editing (Equal). Bouten, Katrien: Formal analysis (Equal), Investigation (Equal), Methodology (Equal). Robouch, Piotr: Conceptualization (Equal), Data curation (Equal), Writing—review & editing (Equal). Emons, Hendrik: Conceptualization (Equal), Supervision (Equal), Writing—review & editing (Equal). Stroka, Joerg: Conceptualization (Equal), Methodology (Equal), Supervision (Equal), Writing—review & editing (Equal).

###  

**Contributors:** M. Adam, P. Alvito, M. Audeon, A. Bahlmann, T. Bessaire, B. Brand, C. Brera, A. Dach, F. Heydebreck, J. Keegan, M. Kolmonen, P. Kormali, I. Lederer, S. MacDonald, H. Mol, G. Moris, I. Pecorelli, I. Pugajeva, L. Reinhold, M. Schneider, M. Solfrizzo, A. Starski, K. Tolzin-Banasch, B. Warth.

## Supplementary Material

qsab094_Supplementary_Data
