# Supplementary material for: Determination of Alternaria Toxins in Tomato, Wheat, and Sunflower Seeds by SPE and LC-MS/MS—A Method Validation Through a Collaborative Trial
Source: J AOAC Int. 2021 Jul 22;105(1):80–94. doi: 10.1093/jaoacint/qsab094 (PMC8824793; doi:10.1093/jaoacint/qsab094)
Supplement: qsab094_Supplementary_Data [file qsab094_supplementary_data.zip › aoac-21-0123-File006.docx]

Table S1 – Data of the homogeneity and stability studies

Excel file

Table S2 – Data submitted by the participants

Excel file

Table S3 – Consistency checking of the data submitted by the laboratories based on the Mandel's *h* and *k* statistics. Yellow – exceeds the Mandel’s indicator at the 5 % significance level. Red – exceeds the Mandel’s indicator at the 1 % significance level.

| TeA:  LC0002 | *h* – 7/15 red ; 1/15 yellow  *k* – 4/11 red ; 2/11 yellow |
| --- | --- |
| ALT:  LC0012 | *h* – 3/7 red ; 1/7 yellow  *k* – 4/7 red |
| AOH:  LC0002 | *h* – 5/13 red ; 1/13 yellow  *k* – 5/8 red ; 1/8 yellow |
| LC0005 | *k* – 5/14 red ; 2/14 yellow |
| TEN:  LC0005 | *h* – 4/15 red ; 1/15 yellow  *k* – 6/14 red ; 1/14 yellow |
| AME:  LC0012 | *h* – 5/15 red  *k* – nothing to highlight |
