# Supplementary material for: Determination of Alternaria Toxins in Tomato, Wheat, and Sunflower Seeds by SPE and LC-MS/MS—A Method Validation Through a Collaborative Trial
Source: J AOAC Int. 2021 Jul 22;105(1):80–94. doi: 10.1093/jaoacint/qsab094 (PMC8824793; doi:10.1093/jaoacint/qsab094)
Supplement: qsab094_Supplementary_Data [file qsab094_supplementary_data.zip › aoac-21-0123-File007.docx]

Figure S1A – Plot of the data collected for ALT normalized to the respective target value. Yellow shaded cells indicate a HorRat > 1.

|  | # 1 | # 2 | # 3 | # 4 | # 5 |
| --- | --- | --- | --- | --- | --- |
| Tomato puree |  |  |  |  |  |
| Wheat |  |  |  |  |  |
| Sunflower |  |  |  |  |  |

Figure S1B – Plot of the data collected for AOH normalized to the respective target value.

|  | # 1 | # 2 | # 3 | # 4 | # 5 |
| --- | --- | --- | --- | --- | --- |
| Tomato puree |  |  |  |  |  |
| Wheat |  |  |  |  |  |
| Sunflower |  |  |  |  |  |

Figure S1C – Plot of the data collected for AME normalized to the respective target value

|  | # 1 | # 2 | # 3 | # 4 | # 5 |
| --- | --- | --- | --- | --- | --- |
| Tomato puree |  |  |  |  |  |
| Wheat |  |  |  |  |  |
| Sunflower |  |  |  |  |  |

Figure S1D – Plot of the data collected for TeA normalized to the respective target value

|  | # 1 | # 2 | # 3 | # 4 | # 5 |
| --- | --- | --- | --- | --- | --- |
| Tomato puree |  |  |  |  |  |
| Wheat |  |  |  |  |  |
| Sunflower |  |  |  |  |  |

Figure S1E – Plot of the data collected for TEN normalized to the respective target value

|  | # 1 | # 2 | # 3 | # 4 | # 5 |
| --- | --- | --- | --- | --- | --- |
| Tomato puree |  |  |  |  |  |
| Wheat |  |  |  |  |  |
| Sunflower |  |  |  |  |  |
